# Rab40 GTPases regulate AMBRA1-mediated transcription and cell migration

**DOI:** 10.1242/jcs.263707

**Published:** 2025-04-11

**Authors:** Revathi Sampath, Katherine Vaeth, Valeryia Mikalayeva, Vytenis Arvydas Skeberdis, Rytis Prekeris, Ke-Jun Han

**Affiliations:** ^1^ The Laboratory of Cell Culture, Lithuanian University of Health Sciences, Kaunas, 50103, Lithuania; ^2^ Department of Cell and Developmental Biology, University of Colorado Anschutz Medical Campus, Aurora, CO 80045, USA

**Keywords:** Rab40 GTPase, AMBRA1, Ubiquitylation, Cell migration

## Abstract

The Rab40 subfamily of proteins consists of unique small monomeric GTPases that form CRL5-based ubiquitin E3 ligase complexes and regulate ubiquitylation of specific target proteins. Recent studies have shown that Rab40 proteins play an important role in regulating cell migration, but the underlying mechanisms of how the Rab40–CRL5 complex functions are still not fully understood. In this study, we identified AMBRA1 as a novel binding partner of Rab40 GTPases and show that this interaction mediates a bidirectional crosstalk between the CRL4 and CRL5 E3 ligases. Importantly, we found that Rab40–CRL5 ubiquitylates AMBRA1, which does not result in AMBRA1 degradation but, instead, appears to induce AMBRA1-dependent regulation of gene transcription. The global transcriptional profiles identified by RNA sequencing showed that AMBRA1 regulates transcription of genes related to cell adhesion and migration. Additionally, we show that AMBRA1-dependent transcription regulation does not require the enzymatic activity of AMBRA1–CRL4, and that Rab40-induced AMBRA1 ubiquitylation leads to dissociation of the AMBRA1–CRL4 complex. Taken together, our findings reveal a novel function of the Rab40–CRL5 complex as an important regulator of AMBRA1-dependent transcription of genes involved in cell migration.

## Teaser

**Summary:** Rab40 GTPases interact with AMBRA1 and cause its non-degradative ubiquitylation. AMBRA1 regulates cancer cell migration, and RNA sequencing reveals global gene profiles regulated by AMBRA1.

## INTRODUCTION

Rab proteins are small monomeric GTPases belonging to the Ras GTPase superfamily. Rab GTPases are evolutionarily conserved and function as key regulators of eukaryotic membrane trafficking. The human genome encodes over 70 Rab GTPases, which can be divided into ten major subfamilies (Homma et al., 2021; Klopper et al., 2012; Leithe et al., 2009; Porfirio-Sousa et al., 2022). Among them, the Rab40 subfamily is unique because it has an extended C-terminal, which contains a suppressor of cytokine signaling (SOCS) box motif (Lee et al., 2007; Neumann and Prekeris, 2023), and thus mediates interaction with cullin-5 (CUL5) to form an E3 ubiquitin ligase complex (Rab40–CRL5) (Duncan et al., 2021; Lee et al., 2007; Neumann and Prekeris, 2023). Therefore, Rab40 proteins function not only as molecular regulators of membrane traffic, but also as part of a ubiquitin E3 ligase complex to mediate ubiquitylation of target proteins (Duncan et al., 2021; Lee et al., 2007; Neumann and Prekeris, 2023).

The Rab40 subfamily consists of four closely related proteins: Rab40a, Rab40al, Rab40b and Rab40c (Duncan et al., 2021; Homma et al., 2021; Neumann and Prekeris, 2023). We and others previously demonstrated that Rab40a and Rab40b are required for regulating cancer cell migration and invasion by promoting extracellular matrix degradation, the dynamics of focal adhesion (FA) sites and invadopodia formation (Dart et al., 2015; Duncan et al., 2022; Han et al., 2022; Jacob et al., 2013, 2016; Linklater et al., 2021). Specifically, Rab40a was reported to mediate proteasomal degradation of RhoU, whereas Rab40b ubiquitylates Eplin and Rap2, thus promoting cell migration by altering FA dynamics and stress fiber formation (Dart et al., 2015; Duncan et al., 2022; Linklater et al., 2021). Additionally, we have shown that Rab40c binds the protein phosphatase 6 (PP6) complex and ubiquitylates the ANKRD28 subunit, thus leading to its lysosomal degradation, which ultimately also affects FAs (Han et al., 2022). All these findings suggest that the Rab40 subfamily of GTPases might have evolved to regulate actin dynamics and FA turnover by mediating ubiquitylation of a specific subset of proteins; however, it remains to be fully understood how Rab40 function is regulated and what molecular machinery governs Rab40-dependent cell migration and invasion.

Our recent proteomics screen identified activating molecule in beclin-1-regulated autophagy (AMBRA1) as a putative target for Rab40c–CRL5-dependent ubiquitylation (Han et al., 2022). AMBRA1 is a WD40 domain-containing protein and is involved in various biological processes including autophagy and cell division (Chaikovsky et al., 2021b; Di Rita and Strappazzon, 2017; Fimia et al., 2013; Nazio et al., 2013; Qin et al., 2022). AMBRA1 acts as a substrate-recognition component of a cullin-4 (CUL4)–DDB1 E3 ubiquitin protein ligase complex (AMBRA1–CRL4), promoting the ubiquitylation of beclin-1 and ULK1, and therefore is a key regulator of autophagy (Fimia et al., 2013, 2011, 2007; Li et al., 2022; Nazio et al., 2013; Qin et al., 2022). Interestingly, it was suggested that AMBRA1 can also mediate crosstalk between cullin-4- and cullin-5-dependent E3 ubiquitin ligases (CRL4 and CRL5) (Antonioli et al., 2014; Chen et al., 2018). Under normal conditions, AMBRA1 binds to CRL4 and is targeted for proteasomal degradation, presumably a consequence of CRL4-dependent auto-ubiquitylation. It was suggested that, upon activation of the autophagy, AMBRA1 inhibits CRL5 activity either by disruption of the interaction between elongin-B and cullin-5 (part of the CRL5 complex) or by mediating proteasomal degradation of elongin-C (Antonioli et al., 2014; Chen et al., 2018). However, it currently remains unclear how this CRL4 and CRL5 crosstalk is regulated and whether AMBRA1 has other CRL4-independent functions.

In addition to its role in autophagy, recent research highlights the important role of AMBRA1 in cancer cell migration and proliferation. AMBRA1 has been proposed to be a tumor suppressor, and loss of AMBRA1 promotes cancer cell growth and invasion (Cianfanelli et al., 2015b; Di Leo et al., 2021; Nazio et al., 2021; Qin et al., 2022). The AMBRA1–CRL4 complex binds to cyclin D, leading to cyclin D ubiquitylation and subsequent proteasomal degradation, thereby controlling the G1-to-S transition and cell division (Chaikovsky et al., 2021a,b; Maiani et al., 2021a,b; Simoneschi et al., 2021). AMBRA1 also regulates Src activity and Src–focal adhesion kinase (FAK)-mediated cancer cell invasion and migration (Di Leo and De Zio, 2021; Schoenherr et al., 2017). AMBRA1 can be recruited to FAs, where it was suggested to bind to both FAK and Src, and AMBRA1 removes active phospho-Src from FAs and transports it into autophagic structures, likely for degradation (Di Leo and De Zio, 2021; Schoenherr et al., 2017).

As our recent work suggested that AMBRA1 interacts with Rab40c, in this study, we decided to investigate whether the interaction between Rab40c and AMBRA1 is a new potential regulatory crosstalk pathway between CRL4 and CRL5 complexes. Consistent with this hypothesis, we show that AMBRA1 enhanced Rab40c binding to cullin-5, and that depletion of AMBRA1 increased Rab40c mRNA and protein levels. We also found that loss of AMBRA1 in MDA-MB-231 cells altered FA distribution and promoted cell migration. Intriguingly, at least some of the AMBRA1 effects on cell adhesion and migration appear to be mediated by AMBRA1-dependent regulation of expression of a subset of genes. Using *SNAI2* as an example, we showed that AMBRA1-dependent transcriptional regulation is independent of AMBRA1 binding to CRL4, but it appears to be regulated by Rab40–CRL5-induced ubiquitylation. Thus, we uncovered a new CRL4-independent function of AMBRA1 in regulating cell migration by regulating gene expression.

## RESULTS

### Rab40c is an AMBRA1-binding protein

**Fig. 1.**
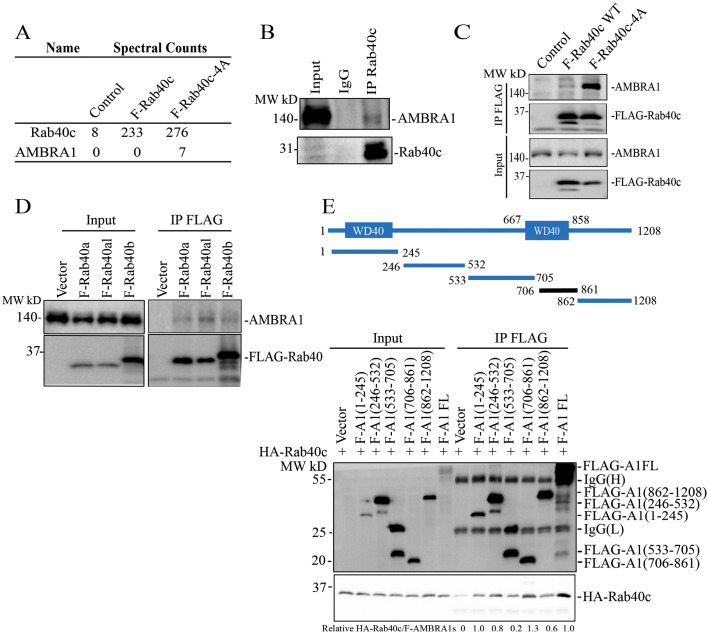
**Rab40 subfamily of small monomeric GTPases interact with AMBRA1.** (A) AMBRA1 co-immunoprecipitates with FLAG–Rab40c-4A in MDA-MB-231 cells. (B) Rab40c was immunoprecipitated from MDA-MB-231 lysates using anti-Rab40c antibodies. The immunoprecipitates were then blotted with anti-AMBRA1 and anti-Rab40c antibodies. (C) 293T cells were transfected with empty plasmid (control), FLAG–Rab40c or FLAG–Rab40c-4A plasmids, and then FLAG–Rab40c [wild-type (WT) or the 4A mutant] was immunoprecipitated with an anti-FLAG antibody. The precipitates were then blotted with anti-AMBRA1 and anti-FLAG antibodies. (D) 293T cells were transfected with empty vector (control), FLAG–Rab40a, FLAG–Rab40al or FLAG–Rab40b plasmids, and then FLAG-tagged Rab40a, Rab40al and Rab40b were immunoprecipitated with an anti-FLAG antibody. Cell lysates and precipitates were then blotted with anti-AMBRA1 or anti-FLAG antibodies. (E) Top: schematic diagram of AMBRA1 deletion mutants. Bottom: 293T cells were co-transfected with HA–Rab40c and empty vector or one of the FLAG-tagged AMBRA1 deletion mutants or the full-length (FL) construct. The FLAG-tagged AMBRA1 proteins were then immunoprecipitated with an anti-FLAG antibody and precipitates were blotted with anti-FLAG and anti-HA antibodies. Immunoblots are representative of three independent experiments.

All proteins of the Rab40 subfamily (Rab40a, Rab40b and Rab40c) regulate cell migration by forming cullin-5-containing Rab40–CRL5 complexes and mediating ubiquitylation of the specific target proteins (Dart et al., 2015; Duncan et al., 2021; Lee et al., 2007; Neumann and Prekeris, 2023). Recent studies from our and other laboratories have demonstrated that Rab40a and Rab40b are both required for cell migration and function by ubiquitylating Eplin, Rap2 and RhoU (Dart et al., 2015; Duncan et al., 2022; Linklater et al., 2021). In contrast, how Rab40c regulates cell migration remains largely unclear. Thus, in this study, we set out to identify new Rab40c–CRL5 substrates. To this end, we developed a proteomics-based screen that relies on our previous studies showing that mutating the 211-LPLP-216 domain (to 211-AAAA-216; termed ‘4A’) within the SOCS box of Rab40 subfamily members (FLAG–Rab40-4A) led to the decrease in cullin-5 binding and an increase in Rab40 association with its ubiquitylation substrates (Han et al., 2022). Among these putative Rab40c–CRL5 substrate proteins, AMBRA1 was present in FLAG–Rab40c-4A but not in FLAG–Rab40c immunoprecipitates (Fig. 1A) (Han et al., 2022). To confirm that AMBRA1 binds to Rab40c, we immunoprecipitated endogenous Rab40c from MDA-MB-231 cells and found that endogenous AMBRA1 co-immunoprecipitated with Rab40c (Fig. 1B). To further confirm the Rab40c and AMBRA1 interaction, we overexpressed either FLAG–Rab40c or FLAG–Rab40c-4A in 293T cells, followed by precipitation with anti-FLAG antibodies and immunoblotting for endogenous AMBRA1. Consistent with our proteomics data, we found that AMBRA1 predominantly binds to FLAG–Rab40c-4A, although some AMBRA1 could also be detected in FLAG–Rab40c wild-type (WT) immunoprecipitates (Fig. 1C). We next tested whether AMBRA1 can also interact with other Rab40 GTPase subfamily members. As shown in Fig. 1D, FLAG-tagged Rab40a, Rab40al and Rab40b all co-immunoprecipitated with endogenous AMBRA1, suggesting that AMBRA1 can interact with all the members of the Rab40 subfamily.

We next set out to map which region of AMBRA1 is responsible for Rab40 binding. To this end, we generated a series of FLAG-tagged AMBRA1 deletion mutants including AMBRA1(1–245), AMBRA1(246–532), AMBRA1(533–705), AMBRA1(706–861) and AMBRA1(862–1208) (Fig. 1E), then individually co-transfected all these constructs with HA–Rab40c into 293T cells, followed by immunoprecipitation with anti-FLAG antibodies. As shown in Fig. 1E, HA–Rab40c predominately co-precipitated with AMBRA1(706–861), which partially overlaps with the C-terminal WD40 domain of the protein (Liu et al., 2023).

### AMBRA1 suppresses Rab40c expression but stimulates Rab40–CRL5 complex formation

**Fig. 2.**
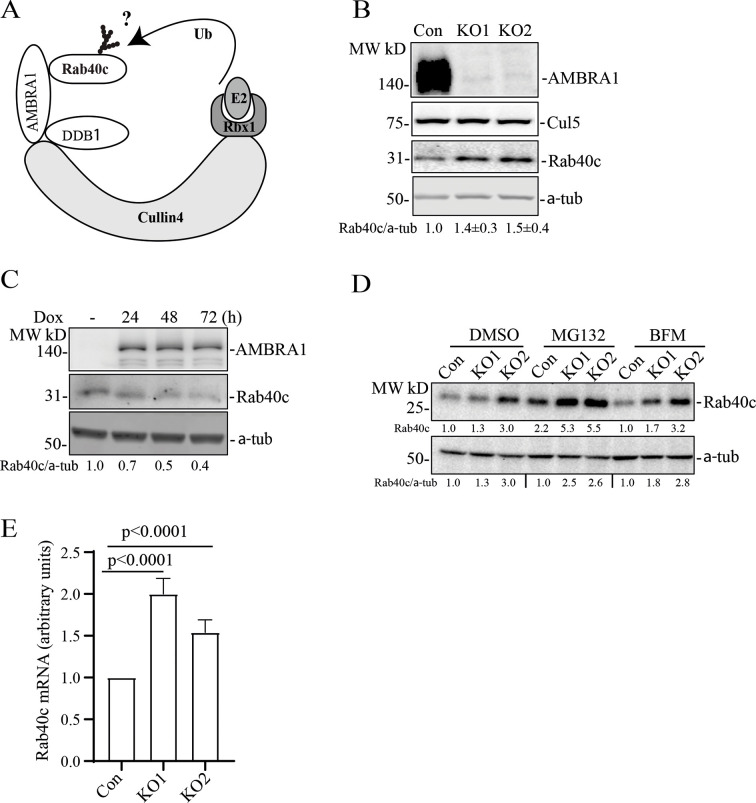
**AMBRA1 regulates Rab40c expression.** (A) A proposed model showing that Rab40c is a putative substrate of the CRL4–AMBRA1 E3 ligase. Ub, ubiquitin. (B) Western blotting analysis of cell lysates from control and AMBRA1-KO cells using the indicated antibodies. Relative Rab40c levels in AMBRA1-KO cells were normalized to loading control and control cells. The numbers shown below the blot are the mean±s.e.m. derived from three independent experiments. (C) AMBRA1-KO cells expressing AMBRA1 under a tetracycline-inducible (Tet-On) promoter were incubated with 100 ng/ml doxycycline (dox) for various time periods and the cell lysates were blotted using indicated antibodies. The numbers shown below the blot are relative amounts of Rab40c normalized to the loading control α-tubulin. Blots represent three independent experiments. (D) Control and AMBRA1-KO cells were treated with DMSO, MG132 and bafilomycin A1 (BFM). The whole-cell lysates were then analyzed by western blotting using the indicated antibodies. The numbers shown below the blot are relative amounts of Rab40c normalized to the control samples. Blots represent three independent experiments. (E) qRT-PCR analysis of *RAB40C* mRNA levels in control and AMBRA1-KO MDA-MB-231 cells. The data shown are the mean±s.e.m. derived from three independent biological replicates. Statistical analysis was performed using two-tailed unpaired Student's *t*-test.

AMBRA1 is a well-established substrate receptor of the cullin-4 ubiquitin ligase complex, which targets protein ubiquitylation and subsequent degradation (Baek and Jang, 2021; Chaikovsky et al., 2021b; Cianfanelli et al., 2015a; Fimia et al., 2007; Jin et al., 2006). Therefore, we hypothesized that AMBRA1 also ubiquitylates Rab40c, leading to its proteasomal degradation (Fig. 2A). To test this, we first generated AMBRA1 knockout (KO) MDA-MB-231 cell lines (KO1 and KO2) using CRISPR/Cas9-mediated genome editing, which were validated by genomic sequencing and western blotting (Fig. S1B; Fig. 2B,C). AMBRA1 KO caused an increase in Rab40c protein levels, whereas it had no effect on the total levels of cullin-5 (Fig. 2B). To further confirm that the increase in Rab40c protein levels is caused by depletion of AMBRA1, we re-introduced AMBRA1, the expression of which is driven by a doxycycline (dox)-inducible promoter, back into an AMBRA1-KO cell line. As shown in Fig. 2C, Rab40c protein levels gradually decreased in cells expressing AMBRA1, suggesting that Rab40c protein level changes are AMBRA1 dependent. Next, we investigated the mechanisms by which AMBRA1 regulates Rab40c protein levels by examining whether AMBRA1 targets Rab40c for proteasomal or lysosomal degradation. To this end, we treated control and AMBRA1-KO cells with either the proteasomal inhibitor MG132 or the lysosomal inhibitor bafilomycin A1 (BFM). As shown in Fig. 2D, MG132 treatment, but not BFM treatment, increased protein levels of Rab40c in both control and AMBRA1-KO cells, suggesting that Rab40c can be degraded by the proteasome pathway. However, the difference in Rab40c protein levels between control and AMBRA1-KO cells did not change substantially after MG132 treatment compared with those in DMSO-treated controls. Therefore, the inhibition of proteasomal degradation is insufficient to block AMBRA1-induced increase in Rab40c protein levels. Based on these data, we hypothesized that AMBRA1 affects transcription of Rab40c mRNA. Consistent with this hypothesis, we found that Rab40c mRNA levels in AMBRA1-KO cells were significantly increased as quantified by real-time quantitative PCR (qRT-PCR) (Fig. 2E).

**Fig. 3.**
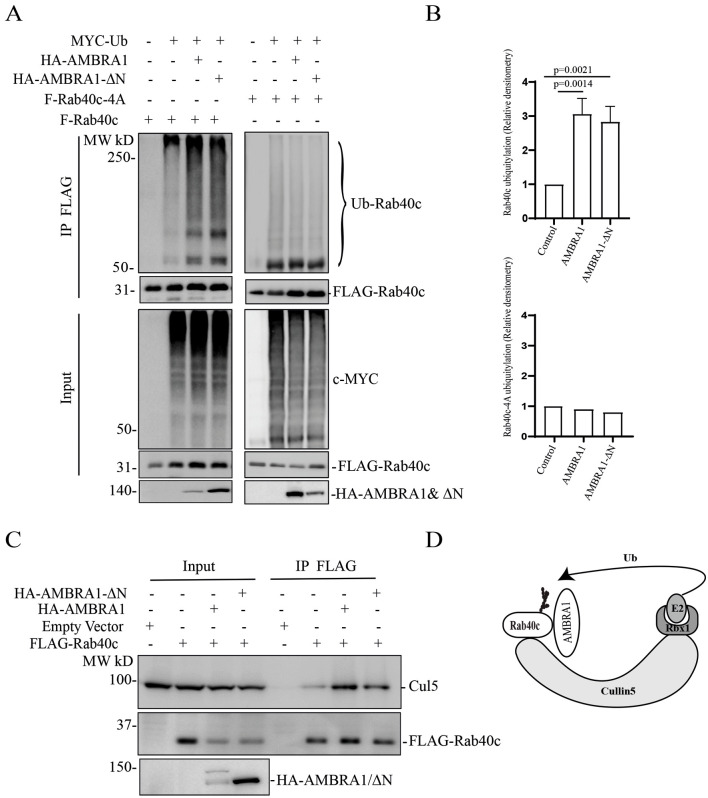
**AMBRA1 regulates Rab40c binding to cullin-5 and its auto-ubiquitylation.** (A) *In vivo* Rab40c ubiquitylation assay. 293T cells were transfected with the indicated plasmids and incubated for 48 h. After being treated with 10 µm MG132 for 6 h, cells were harvested and immunoprecipitated with an anti-FLAG antibody, followed by western blotting with anti-Myc, anti-FLAG and anti-HA antibodies. The Myc signal in anti-FLAG immunoprecipitates represents the extent of FLAG–Rab40c ubiquitylation. (B) Quantification of the levels of Rab40c polyubiquitylation shown in panel A. The data shown are the mean±s.e.m. derived from three different independent experiments. Statistical analysis was performed using two-tailed unpaired Student's *t*-test. (C) 293T cells were co-transfected with the indicated plasmids. FLAG-tagged Rab40c was then immunoprecipitated with the anti-FLAG antibody and precipitates were immunoblotted with the indicated antibodies. Blots represent three independent experiments. (D) A proposed model showing that AMBRA1 promotes Rab40c auto-ubiquitylation.

** **As AMBRA1 binds to cullin-4 and acts as a substrate receptor for the AMBRA1–CRL4 complex (Fig. 2A), we next examined whether AMBRA1–CRL4 can regulate Rab40c polyubiquitylation. To this end, we transfected 293T cells with constructs encoding FLAG–Rab40c, Myc–ubiquitin (Ub), HA–AMBRA1 and HA–AMBRA1-ΔN (an AMBRA1 mutant lacking residues 1–20 that does not bind cullin-4) individually or in various combinations (Fig. 3A). Lysates were then immunoprecipitated with anti-FLAG antibodies and blotted for Myc–Ub with anti-Myc antibodies. When the Myc–Ub construct was co-transfected with the FLAG–Rab40c construct in the presence of the proteasomal inhibitor MG132, high-molecular mass species were detected, presumed to be polyubiquitylated Rab40c, which were significantly enhanced in samples co-transfected with the construct encoding HA–AMBRA1. Surprisingly, similar to HA–AMBRA1 WT, HA–AMBRA1-ΔN also stimulated Rab40c polyubiquitylation (Fig. 3A,B), suggesting that AMBRA1–CRL4 ligase activity is not required for Rab40c ubiquitylation. We speculate that AMBRA1 binding to Rab40c promotes auto-ubiquitylation of Rab40c by the Rab40c–CRL5 complex. To test this hypothesis, we performed a similar ubiquitylation assay as described above, except that FLAG–Rab40c was replaced by FLAG–Rab40c-4A, a mutant that does not bind to cullin-5 and cannot be part of CRL5 complex (Han et al., 2022). As shown in Fig. 3A,B, FLAG–Rab40c-4A ubiquitylation was barely detected even with co-transfection with the Myc–Ub construct, suggesting that Rab40c-4A lost self-catalyzed ubiquitylation. Under these conditions, co-transfection with either the HA–AMBRA1 WT or HA–AMBRA1-ΔN constructs no longer had any effect on Rab40c-4A ubiquitylation (Fig. 3A,B). Taken together, these results demonstrate that AMBRA1 binding, but not AMBRA1–CRL4 ubiquitin ligase activity, is necessary for Rab40c ubiquitylation. Our data raise the possibility that AMBRA1 enhances Rab40c self-ubiquitylation by regulating Rab40c interaction with cullin-5. To test this, we overexpressed FLAG–Rab40c individually or with HA–AMBRA1 or HA–AMBRA1-ΔN, followed by precipitation with anti-FLAG antibodies and immunoblotting for endogenous cullin-5. As shown in Fig. 3C, FLAG–Rab40c could co-immunoprecipitate with endogenous cullin-5, as we reported previously (Han et al., 2022). Importantly, the amount of cullin-5 co-immunoprecipitating with FLAG–Rab40c substantially increased when FLAG–Rab40c was co-transfected either with HA–AMBRA1 or HA–AMBRA1-ΔN (Fig. 3C). Therefore, we propose that AMBRA1 binding can increase Rab40c interaction with cullin-5 and results in Rab40c self-ubiquitylation and activation independent of AMBRA1–CRL4 ubiquitin E3 ligase activity (Fig. 3D).

### AMBRA1 binding to cullin-4 is regulated by Rab40–CRL5-dependent ubiquitylation

**Fig. 4.**
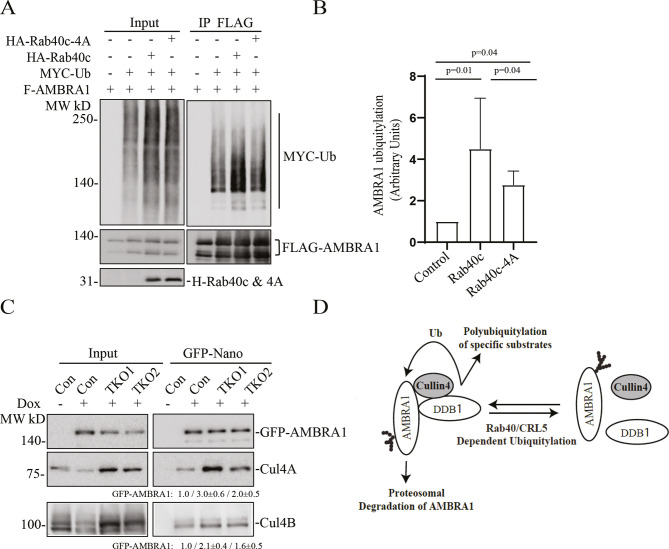
**Rab40c–CRL5 ubiquitylates AMBRA1.** (A) *In vivo* AMBRA1 ubiquitylation assay. 293T cells were transfected with the indicated plasmids and incubated for 24 h. After treatment with 100 nm BFM overnight, cells were harvested and immunoprecipitated with an anti-FLAG antibody, followed by western blotting using anti-Myc, anti-FLAG or anti-HA antibodies. The Myc signal in anti-FLAG immunoprecipitates represents the extent of FLAG–AMBRA1 ubiquitylation. (B) Quantification of FLAG–AMBRA1 ubiquitylation from panel A. The data shown represent the mean±s.e.m. derived from three independent experiments and are normalized against the control. Statistical analysis was performed using two-tailed unpaired Student's *t*-test. (C) Control MDA-MB-231 cells and Rab40a, Rab40b and Rab40c triple-knockout MDA-MB-231 cells stably expressing dox-inducible GFP–AMBRA1 were incubated with 100 ng/ml dox for 48 h. GFP–AMBRA1 was then immunoprecipitated using an anti-GFP nanobody and immunoprecipitates were immunoblotted with the indicated antibodies. The quantification shown represents mean±s.e.m. derived from three independent experiments and normalized against the control. (D) A proposed model showing that Rab40–CRL5 ubiquitylates AMBRA1 and inhibits AMBRA1–CRL4 complex formation.

Because the Rab40 subfamily of proteins are substrate receptors for the CRL5 complex (Duncan et al., 2021; Lee et al., 2007; Neumann and Prekeris, 2023; Zhao et al., 2020), we next tested whether Rab40 can ubiquitylate and regulate AMBRA1. To this end, we transfected 293T cells with constructs encoding FLAG–AMBRA1, Myc–Ub, HA–Rab40c or HA–Rab40c-4A (the Rab40c mutant that does not bind cullin-5) individually or in various combinations (Fig. 4A). Lysates were then immunoprecipitated with anti-FLAG antibodies and blotted for Myc–Ub with anti-Myc antibodies. When the Myc–Ub construct was co-transfected with the FLAG–AMBRA1 construct in the presence of the proteasomal inhibitor MG132, high-molecular mass species were detected, presumed to be polyubiquitylated AMBRA1. AMBRA1 polyubiquitylation was enhanced by co-transfecting the HA–Rab40c construct (Fig. 4A,B). Importantly, co-transfecting the construct expressing the cullin-5-binding-deficient Rab40c mutant (HA–Rab40c-4A) decreased Rab40c-induced increase in AMBRA1 ubiquitylation. Note that HA–Rab40c-4A did not completely block AMBRA1 ubiquitylation (Fig. 4A,B), suggesting that AMBRA1 can also be ubiquitylated by other E3 ligases. Indeed, it was previously reported that AMBRA1 can self-ubiquitylate (as well as ubiquitylate other substrates) by forming the CRL4 complex with cullin-4 and DDB1 (Fig. 4D) (Antonioli et al., 2014).

Next, we set out to determine the functional consequences of AMBRA1 ubiquitylation by Rab40–CRL5. Given that AMBRA1 can interact with all members of Rab40 subfamily, we used Rab40a, Rab40b and Rab40c triple-knockout (TKO) MDA-MB-231 cells (Linklater et al., 2021) for the rest of the study. The best-described AMBRA1 function is in the formation of the AMBRA1–CRL4 complex that mediates polyubiquitylation and degradation of several proteins involved in cell proliferation and autophagy (Chaikovsky et al., 2021a; Chen et al., 2018; Cianfanelli et al., 2015a,b; Di Bartolomeo et al., 2010; Maiani et al., 2021a; Simoneschi et al., 2021). Thus, we next tested the effect of Rab40-TKO on the formation of the AMBRA1–CRL4 complex. To this end, we generated three different cell lines (control, TKO1 and TKO2) stably expressing dox-inducible GFP–AMBRA1. GFP–AMBRA1 was then immunoprecipitated using an anti-GFP nanobody and immunoblotted for the presence of cullin-4. As shown in Fig. 4C, the depletion of Rab40 increased cullin-4A (CUL4A) and cullin-4B  (CUL4B) association with GFP–AMBRA1. Altogether, these results suggest that Rab40–CRL5-dependent ubiquitylation of AMBRA1 leads to disassembly of the AMBRA1–CRL4 complex (Fig. 4D).

### AMBRA1 regulates transcription

**Fig. 5.**
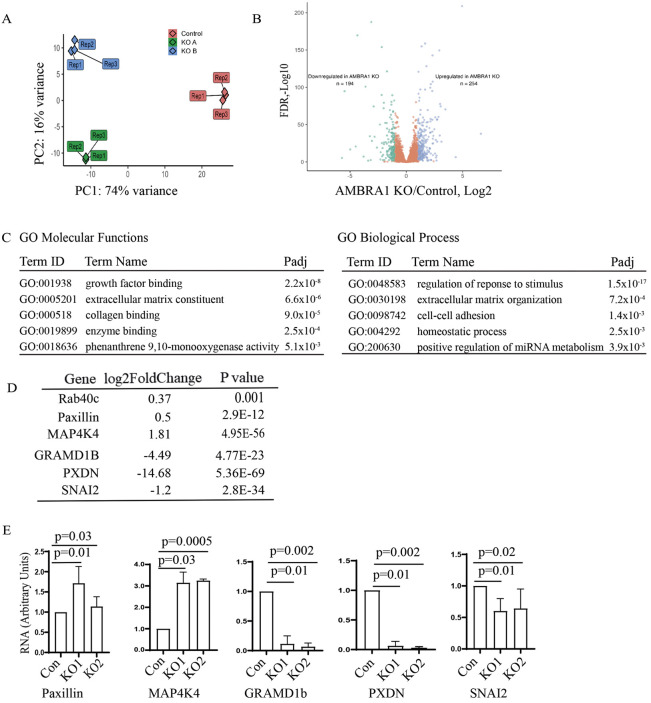
**AMBRA1 regulates gene transcription.** (A) Principal component analysis representing the differences between the control (three replicates) and the AMBRA1-KO (two different AMBRA1-KO cell lines, each with three replicates) RNA-seq datasets. (B) Volcano plot showing the significantly downregulated (green) and significantly upregulated (blue) mRNAs identified in AMBRA1-KO cells. FDR, false discovery rate. (C) Gene Ontology (GO) molecular functions and biological process enrichment analysis of significantly changed mRNAs (upregulated and downregulated) identified by RNA-seq. (D,E) qPCR analysis of selected mRNAs that are either increased or decreased in AMBRA1-KO cells as indicted by RNA-seq. The mean±s.d. were calculated from three independent experiments. Statistical analysis was performed using two-tailed unpaired Student's *t*-test.

**Fig. 6.**
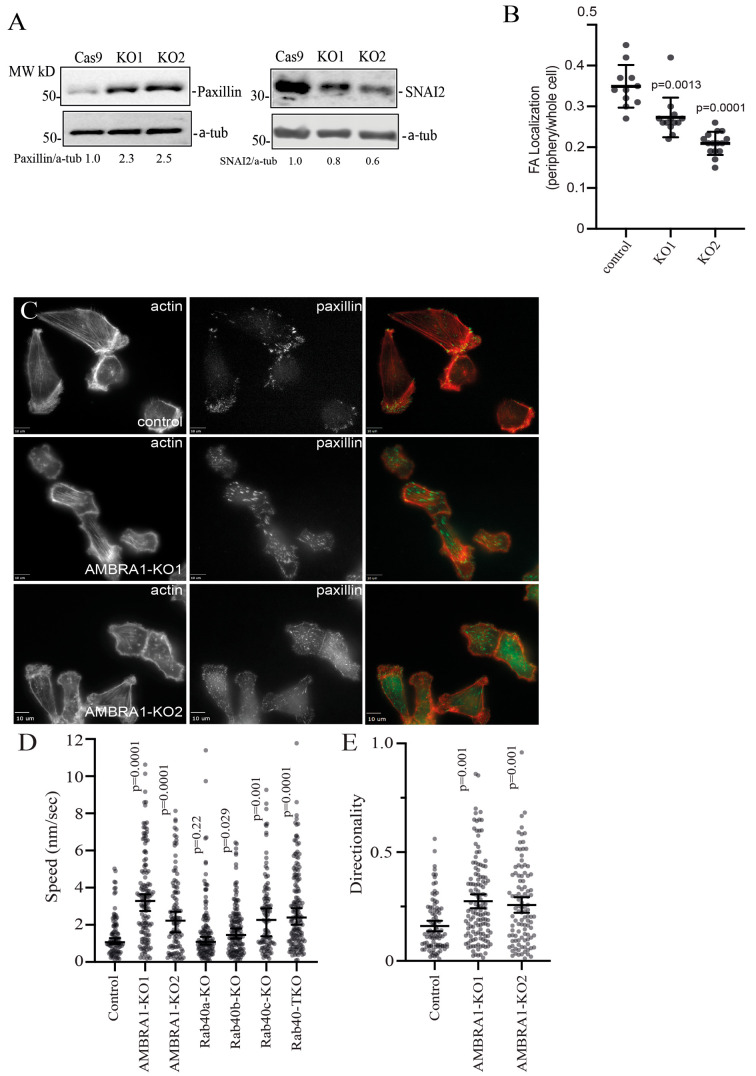
**AMBRA1 regulates MDA-MB-231 cell migration.** (A) To measure the levels of paxillin and SNAI2, cell lysates from control and AMBRA1-KO cells were probed with anti-paxillin and anti-SNAI2 antibodies. α-tubulin was used as a loading control. Blots represent three independent experiments. (B) Quantification of the ratio of focal adhesions (FAs) at the cell periphery (within 4 µm from plasma membrane) to FAs in the whole cells for control and AMBRA1-KO cells. Data shown are the mean±s.d. derived from three independent experiments. Statistical analysis was performed using two-tailed unpaired Student's *t*-test. (C) Control, AMBRA1-KO1 and AMBRA1-KO2 MDA-MB-231 cells were plated on collagen-coated coverslips for 24 h. Cells were then fixed and stained with phalloidin–Alexa Fluor 594 (red) to visualize actin and anti-paxillin antibody (green). Scale bars: 10 µm. (D) Migration analysis of control, AMBRA1-KO, Rab40a-KO, Rab40b-KO, Rab40c-KO and Rab40a/Rab40b/Rab40c TKO cells. For migration velocity quantification, 95–150 cells were randomly chosen for velocity tracking, and velocity data (nm/s) were extracted for each individual cell. The data shown are the mean±s.d. derived from three biological replicates. Mann–Whitney test was performed to compare the velocities of cells from different cell lines. (E) Cell directionality was calculated as a ratio of the net displacement of a cell from its starting to final position compared with the total distance traveled from time-lapse analysis. Data shown represent the mean±s.e.m. derived from three different experiments. Mann–Whitney test was performed to compare the velocities of cells from different cell lines.

Our data (see Fig. 2) suggest that AMBRA1 affects Rab40c protein levels by regulating the transcription of *RAB40C* mRNA. This raises the interesting possibility that AMBRA1 has two distinct functions: to mediate protein ubiquitylation and proteasomal degradation as part of the AMBRA1–CRL4 complex, and to regulate gene transcription. Indeed, a recent study reported that AMBRA1 is present in the nucleus where it appears to affect transcription (Schoenherr et al., 2020). What remains unclear is what genes are regulated by nuclear AMBRA1 and whether this regulation of transcription is dependent on AMBRA1–CRL4 ubiquitylation activity. Therefore, to identify the genes that are regulated by AMBRA1, we performed RNA sequencing (RNA-seq) analysis comparing control MDA-MB-231 cells with two different AMBRA1-KO MDA-MB-231 cell lines. Initial principal component analysis demonstrated that AMBRA1-KO RNA samples were similar to each other, indicating the reproducible nature of their RNA content (Fig. 5A). Importantly, AMBRA1-KO RNA samples were well separated from control samples, suggesting that the AMBRA1-KO transcriptome is distinct from that of control cells (Fig. 5A). Compared with control cells, AMBRA1 KO led to downregulation of 194 genes and upregulation of 254 genes [KO/control, log_2_(fold change)>2, *P*<0.05] (Fig. 5B). Gene Ontology functional analysis revealed that many of these genes are involved in cell migration-related processes such as growth factor binding, extracellular matrix composition and organization, collagen binding, and cell–cell adhesion (Fig. 5C). Notably, we found that *RAB40C* mRNA levels were significantly increased in AMBRA1-KO cells (Fig. 5D), consistent with our previous observations (Fig. 2). To further confirm RNA-seq results, we performed quantitative PCR (qPCR) analysis on several selected mRNAs related to cell adhesion and migration, such as paxillin (*PXN*), *MAP4K4*, *GRAMD1B*, *PXDN* and *SNAI2* (Fig. 5D,E). Importantly, an increase in paxillin expression and a decrease in SNAI2 expression was also confirmed by western blotting (Fig. 6A).

### AMBRA1 regulates cell migration

Our transcriptomic analysis indicated that AMBRA1 might be involved in regulating cell migration. For example, RNA-seq, qPCR and western blotting analyses showed that AMBRA1 differently regulates paxillin and SNAI2 transcription and protein levels. Paxillin is a FA adapter that is involved in FA formation and signal transduction, thus regulating cell adhesion and migration (Lopez-Colome et al., 2017; Ripamonti et al., 2022; Schaller, 2001; Turner, 2000). SNAI2 is a snail family transcriptional repressor that is involved in regulating the epithelial-to-mesenchymal transition (EMT) and the migration of cancer cells (Cobaleda et al., 2007; Phillips and Kuperwasser, 2014; Zhou et al., 2019). We therefore investigated whether the cell motility and the structure of FAs changed in AMBRA1-KO cells. First, we assessed the number, size and distribution of FAs in control and AMBRA1-KO cells using an anti-paxillin antibody. As previously reported (Han et al., 2022), in control MDA-MB-231 cells, paxillin-positive dot-like FAs were mostly present at the cell periphery, especially in leading-edge lamellipodia (Fig. 6B,C). In agreement with an increase in paxillin protein levels in AMBRA1-KO cells, an increase in the size (but not number) of paxillin-positive FAs was observed in these cells (Fig. 6B,C; Fig. S2B). Strikingly, FAs did not accumulate at the periphery of the cell but, instead, were scattered throughout whole cells (Fig. 6B,C).

To directly test whether AMBRA1 regulates cell migration, we automatically tracked individual cell movements over time to quantify migration velocity. As shown in Fig. 6D, compared with control cells, AMBRA1-KO cells exhibited increased individual cell migration and velocity. Importantly, MDA-MB-231 TKO cells (lacking Rab40a, Rab40b and Rab40c) exhibited similar phenotypes, suggesting that Rab40 GTPases regulate cell migration, in part, by binding and ubiquitylating AMBRA1. We analyzed the migration directionality of control and AMBRA1-KO cells and found that AMBRA1-KO cells exhibited enhanced directionality during migration (Fig. 6E), supporting the hypothesis that Rab40–AMBRA1 pathway regulates cell migration and FA function.

### Transcriptional regulation by AMBRA1 does not require AMBRA1–CRL4-dependent ubiquitylation

**Fig. 7.**
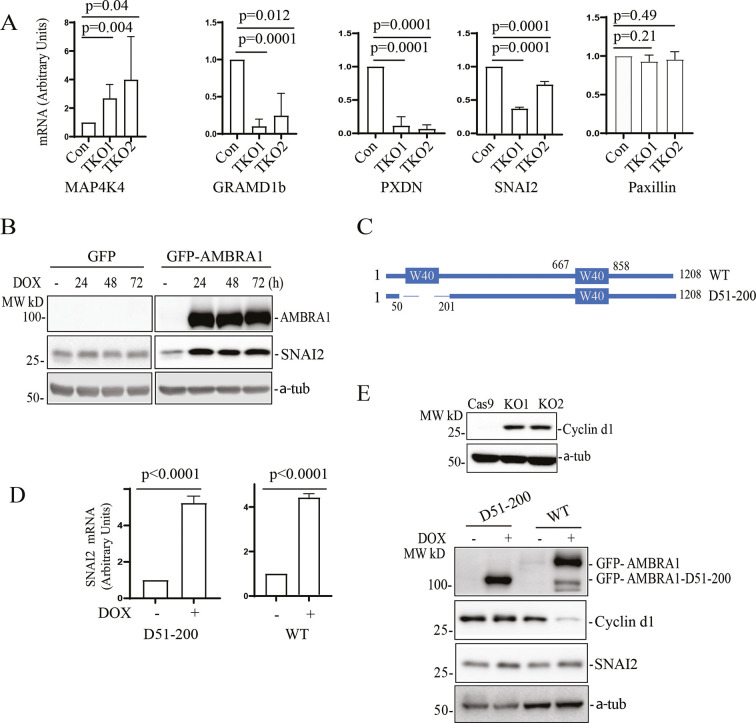
**Transcriptional regulation by AMBRA1 is independent from AMBRA1–CR4 complex formation.** (A) qRT-PCR analysis of selected mRNAs that were affected in AMBRA1-KO cells (see Fig. 5E). RNAs were isolated from control and Rab40-TKO MDA-MB-231 cells. The data shown are the mean±s.d. calculated from three independent experiments. Statistical analysis was performed using two-tailed unpaired Student's *t*-test. (B) AMBRA1-KO MDA-MB-231 cells stably expressing dox-inducible GFP or GFP–AMBRA1 were incubated with 100 ng/ml dox for the indicated times. Cell lysates were then immunoblotted with anti-SNAI2, anti-AMBRA1 and anti-α-tubulin (loading control) antibodies. Blots represent three independent experiments. (C) Schematic representation of AMBRA1 and the AMBRA1 N-terminal WD40 deletion mutant (D51–200). (D) qRT-PCR analysis of the levels of *SNAI2* mRNA in AMBRA1-KO MDA-MB-231 cells stably expressing either dox-inducible GFP–AMBRA1(D51−200) or dox-inducible GFP–AMBRA1. The mean±s.d. were calculated from three independent experiments. Statistical analysis was performed using two-tailed unpaired Student's *t*-test. (E) Top: immunoblotting of cell lysates from control and AMBRA1-KO cells with anti-cyclin D1 and anti-α-tubulin (loading control) antibodies. Bottom: AMBRA1-KO MDA-MB-231 cells stably expressing dox-inducible GFP–AMBRA1(D51−200) or GFP–AMBRA1 were incubated with 100 ng/ml dox for 48 h. Cell lysates were immunoblotted with anti-AMBRA1, anti-SNAI2, anti-cyclin D1 and anti-α-tubulin antibodies. Blots represent three independent experiments.

Our data suggest that Rab40 GTPases bind and ubiquitylate AMBRA1. Furthermore, this Rab40-dependent ubiquitylation appears to regulate AMBRA1 function. Consequently, it would be expected that Rab40 KO and AMBRA1 KO would lead to similar changes in gene expression. To test this hypothesis, we performed qPCR analysis of selected transcripts that were affected in AMBRA1-KO cells (as determined by RNA-seq and qPCR). As shown in Fig. 7A, KO of all three Rab40 isoforms (Rab40 TKO) led to a decrease in *GRAMD1B*, *PXDN* and *SNAI2* expression, as well as an increase in *MAP4K4* expression, the changes that were also observed in AMBRA1-KO cells (Fig. 5). However, paxillin mRNA levels remain unchanged in Rab40-TKO cells (Fig. 7A), suggesting that paxillin mRNA transcription is not regulated by Rab40. Further studies will be needed to determine why some AMBRA1-induced transcriptional changes are regulated by Rab40, whereas others are not. In this study, we focused on mRNAs that are regulated by both AMBRA1 and Rab40.

To determine whether AMBRA1 overexpression can rescue changes in gene expression in Rab40-TKO cells, we made a Rab40-TKO cell line stably expressing dox-inducible AMBRA1 and performed qPCR to analyze *SNAI2*, *MAP4K4*, *GRAMD1B* and *PXDN* mRNA levels. As shown in Fig. S3A, AMBRA1 overexpression had little effect on *MAP4K4*, *SNAI2*, *GRAMD1B* and *PXDN* mRNA levels, suggesting that Rab40 is required for transcriptional AMBRA1 function. Taken together, these data suggest that the Rab40–AMBRA1 pathway is involved in regulating gene transcription.

As SNAI2 is a key transcriptional factor for MDA-MB-231 migration and invasion, we selected it for further investigation. First, we confirmed that GFP–AMBRA1 but not GFP can rescue SNAI2 expression when their expressions were induced by dox in AMBRA1-KO cells (Fig. 7B). Although our data suggest that AMBRA1 regulates gene transcription, the underlying mechanism governing this AMBRA1 function remains unclear. One possibility is that this function is dependent on cullin-4A or cullin-4B, as they have both been reported to localize to the nucleus, where they appear to mediate multiple nucleus-related functions, including DNA replication, DNA repair and chromatin remodeling (Hannah and Zhou, 2015; Hu et al., 2004; Jackson and Xiong, 2009; Wang et al., 2006). Another possibility is that the cytosolic AMBRA1–CRL4 complex ubiquitylates selected transcription regulators, thus affecting their protein levels and/or their translocation to nucleus. AMBRA1 contains an N-terminal WD40 domain that is responsible for binding cullin-4A/cullin-4B and the DDB1 E3 ligase to form a AMBRA1–CRL4 complex. To test whether AMBRA1 transcription regulatory function is dependent on CRL4 binding, we generated two AMBRA1-KO cell lines stably expressing a dox-inducible GFP-tagged WT AMBRA1 or an N-terminal WD40 deletion mutant (D51–200) that cannot bind to cullin-4 (Fig. 7C) (Liu et al., 2023; Nazio et al., 2013). To confirm that the AMBRA1(D51–200) mutant cannot mediate proteasomal degradation, we immunoblotted cell lysates using an anti-cyclin D1 antibody. Cyclin D1 is a known target of AMBRA1–CRL4-dependent ubiquitylation and degradation (Chaikovsky et al., 2021a,b; Lu et al., 2023; Maiani et al., 2021b; Simoneschi et al., 2021). Consistent with previous reports, AMBRA1 KO led to an increase in cyclin D1 protein levels and resulted in a delay in cells entering the G2/M phase (Fig. S2). This AMBRA1-KO induced increase in cyclin D1 proteins levels could be eliminated by overexpressing WT AMBRA1 but not the AMBRA1(D51–200) mutant (Fig. 7E), demonstrating that AMBRA1–CRL4 ubiquitylation activity is needed to regulate cyclin D1 levels. Next, we tested whether AMBRA1–CRL4 ubiquitylation activity is required for an increase in *SNAI2* mRNA. To this end, we measured *SNAI2* mRNA levels in MDA-MB-231 cells by qPCR. Induction of GFP–AMBRA1 expression in AMBRA1-KO cells increased *SNAI2* mRNA levels, which is consistent with our RNA-seq results, suggesting that AMBRA1 is a positive regulator for *SNAI2* expression (Fig. 7D). Intriguingly, *SNAI2* mRNA levels were also significantly increased when AMBRA1(D51–200) expression was induced, suggesting that AMBRA1 activates *SNAI2* transcription independent of AMBRA1–CRL4 complex formation. All these data strongly suggest that, in addition to its function as a component of the E3 ligase complex, AMBRA1 also functions as a transcriptional regulator, which is dependent on its interaction with Rab40 GTPases.

### The roles of different AMBRA1 splice isoforms in regulation of transcription

Our data so far identified a novel function for AMBRA1 as a transcriptional regulator that is not dependent on its ability to bind cullin-4- and CRL4-mediated protein ubiquitylation. What remains unclear is whether AMBRA1 directly regulates transcription by translocating to the nucleus or whether it acts as a cytosolic scaffolding protein that indirectly affects transcription by sequestering other transcriptional regulators in cytosol. Importantly, AMBRA1 exists as several different splice isoforms (Fig. S3B). The main difference between these isoforms is the presence or absence of two insertions, one at amino acid 601 (isoform 1, ISO1) and a second at amino acid 255 (isoform 5, ISO5). We used isoform 2 for all overexpression analyses shown in this study. Thus, we next asked whether these different isoforms have a differential function in regulating protein ubiquitylation/degradation and transcription. To this end, we generated two additional MDA-MB-231 AMBRA1-KO cell lines stably expressing dox-inducible isoforms 1 and 5 of AMBRA1 (Fig. S3C). These cell lines were then tested for their ability to target cyclin D1 for proteasomal degradation. As shown in Fig. S3C, isoforms 1 and 5 could both induce cyclin D1 degradation in a manner similar to isoform 2 (Fig. 7E). Next, we used qPCR to test whether isoforms 1 and/or 5 could increase *SNAI2* expression. As shown in Fig. S3D, all tested AMBRA1 isoforms could stimulate *SNAI2* transcription.

Although our data demonstrate that, when overexpressed, all AMBRA1 splice isoforms can induce *SNAI2* expression and cyclin D1 proteasomal degradation, we wondered whether there are differences in nuclear import between these AMBRA1 isoforms. To this end, we fractionated isoform 1-, 2- and 5-expressing cells into nuclear (N) and cytosolic (C) fractions and compared the distribution of different AMBRA1 isoforms between the cytosol and nucleus. Interestingly, although all isoforms could be detected in the nucleus, isoform 5 appeared to be targeted to the nucleus more efficiently than isoforms 1 and 2 (Fig. S4A). To further confirm AMBRA1 nuclear localization, we next used immunofluorescence microcopy to analyze the subcellular distribution of all three AMBRA1 isoforms. As shown in Fig. S4B, AMBRA1 isoforms 1 and 2 were predominately present in cytosol. In contrast, AMBRA1 isoform 5 could clearly be observed in the nucleus and perinuclear organelles. Thus, although further research is needed to better define the roles of different AMBRA1 splice isoforms, our data suggest that isoform 5 has a distinct subcellular localization and function compared to the other AMBRA1 isoforms.

## DISCUSSION

The Rab40–CRL5 protein complex has recently emerged as a unique Rab40-dependent complex that plays an important role in regulating spatiotemporal dynamics of protein ubiquitylation during cell migration. Intriguingly, in many cases, this ubiquitylation does not induce degradation of target proteins but rather regulates their localization and activity (Duncan et al., 2022; Han et al., 2022; Linklater et al., 2021). In this study, we identified AMBRA1 as a novel Rab40–CRL5 substrate protein and show that the Rab40–CRL5 E3 ligase mediates non-proteolytic polyubiquitylation of AMBRA1. Surprisingly, we found that AMBRA1 ubiquitylated by Rab40–CRL5 appears to function as a transcriptional regulator and that AMBRA1 affects transcription independent of its ability to bind cullin-4 and mediate protein ubiquitylation. Thus, our findings expand the current understanding of the molecular functions of AMBRA1 and suggest that AMBRA1 has at least two distinct functions. The first one is the canonical function of regulating protein polyubiquitylation and degradation during activation of autophagy. The second one is transcriptional regulation of selected subsets of mRNAs, the process that is independent from AMBRA1–CRL4 enzymatic activity.

AMBRA1 is an intrinsically disordered protein that has been shown to bind numerous other proteins and perform many functions. Although we used multiple approaches to establish the Rab40 subfamily of GTPases as novel binding partners of AMBRA1, it remains unclear whether their interaction is direct or mediated by other proteins. Although Rab40 binds both AMBRA1 and CRL5, we could not detect AMBRA1 binding (via Rab40) to CRL5. One possibility is that AMBRA1 is a Rab40–CRL5 substrate and AMBRA1 ubiquitylation would likely cause its rapid dissociation from the Rab40c–cullin-5 complex. Consistent with the idea, we found that the Rab40c-4A mutant (that does not bind cullin-5 and is thus enzymatically inactive) binds to AMBRA1 more strongly than WT Rab40c.

AMBRA1 is a substrate receptor for CRL4, whereas Rab40s act as the adaptor proteins for CRL5; therefore, their interaction might result in ubiquitylation of each other. In fact, we observed that overexpression of AMBRA1 can stimulate Rab40c ubiquitylation. However, AMBRA1-ΔN, a mutant unable to bind DDB1 (Liu et al., 2023), can also stimulate Rab40c ubiquitylation (Fig. 3A), suggesting that Rab40c is not a substrate of CRL4, but rather that their interaction stimulates Rab40c self-ubiquitylation. In support of this, we found that AMBRA1 lost its ability to stimulate ubiquitylation of Rab40c-4A, which cannot bind to cullin-5 and form the Rab40–CRL5 complex (Han et al., 2022). As AMBRA1 appears to increase Rab40c binding to cullin-5, AMBRA1 might function as a positive regulator of Rab40–CRL5 complex assembly, differing from two recent reports showing that AMBRA1 represses CRL5 activity by binding elongin-B or by targeting elongin-C for degradation (Antonioli et al., 2014; Chen et al., 2018). The reason for these different effects of AMBRA1 on CRL5 activity is unclear, but we speculate that AMBRA1 differentially modulates CRL5 signaling during different cellular processes such as autophagy (binding elongin-B), inflammation (degrading elongin-C) or cell migration (promoting Rab40–CRL5 assembly). Interestingly, we found that AMBRA1 is a substrate of Rab40c–CRL5-dependent ubiquitylation. Previous studies have shown that AMBRA1 undergoes post-translational modifications, including cullin-4-dependent self-ubiquitylation of AMBRA1, or RNF2-mediated AMBRA1 polyubiquitylation, leading to proteasomal degradation (Antonioli et al., 2014; Morel et al., 2014; Xia et al., 2014). In this study, we suggest that Rab40–CRL5-mediated AMBRA1 ubiquitylation does not lead to its degradation but instead appears to mediate the disassembly of the AMBRA1–CRL4 complex.

Although Rab40c is not a substrate of AMBRA1–CRL4-dependent ubiquitylation and proteasomal degradation, Rab40c protein levels were substantially increased in AMBRA1-KO cells. Interestingly, we demonstrate that *RAB40C* mRNA levels increase in AMBRA1-KO cells, suggesting that AMBRA1 regulates (directly or indirectly) *RAB40C* transcription. Reminiscent of a recent study in which AMBRA1 was found to bind with multiple classes of proteins in the nucleus to regulate gene transcription (Schoenherr et al., 2020), we set to further dissect the role of AMBRA1 in regulating transcription and to identify the global gene profiles affected by AMBRA1 depletion. The RNA-seq results confirmed that *RAB40C* mRNA did indeed increase in AMBRA1-KO cells. Importantly, AMBRA1 depletion led to changes in the transcription of multiple genes, suggesting that AMBRA1 is a transcriptional regulator of these subsets of genes. How AMBRA1 regulates gene transcription remains unclear and will be a subject of further studies. As AMBRA1 does not have a clearly identifiable DNA-binding domain, it is unlikely that AMBRA1 directly binds to the promoter or enhancer regions. One possibility is that AMBRA1 is recruited to specific promoters by binding chromatin-modifying proteins. Indeed, it was previously suggested that AMBRA1 can regulate histone methylation (Schoenherr et al., 2020). Another possibility is that AMBRA1 regulates the activity of some transcriptional activators or repressors by either directly binding to them (and acting as a scaffolding factor) or mediating their ubiquitylation. For example, a recent study showed that AMBRA1–CRL4 mediates non-proteolytic polyubiquitylation of Smad4 to enhance its transcriptional functions (Liu et al., 2021). Surprisingly, we have shown that AMBRA1-dependent transcriptional regulation of *SNAI2* does not require the AMBRA1–CRL4 E3 ligase complex. This raises the intriguing possibility that AMBRA1 has a non-canonical function in regulating gene transcription independent of CRL4-mediated ubiquitylation. However, further studies will be needed to confirm this and to dissect the molecular machinery governing AMBRA1-dependent regulation of transcription.

It is well established that protein ubiquitylation plays a crucial role in regulating gene transcription (Kodadek, 2010; Mark and Rape, 2021; Ndoja and Yao, 2014). Non-proteolytic polyubiquitylation of AMBRA1 by Rab40–CRL5 might affect AMBRA1-mediated transcription. Consistent with this idea, we found that transcription of some selected genes is affected in AMBRA1 KO and Rab40 TKO cells, suggesting that Rab40 and AMBRA1 co-regulate the same subset of genes. In this regard, Rab40–CRL5 establishes a negative feedback mechanism to regulate itself by ubiquitylating AMBRA1, which then represses (directly or indirectly) Rab40 transcription. Intriguingly, although we show that AMBRA1 regulates transcription, it does not have a canonical nuclear localization signal. Previous studies suggested that CRL4s are localized in the nucleus, especially CRL4B, which contains a nuclear localization signal in its N-terminus (Hannah and Zhou, 2015). However, we found that AMBRA1-dependent transcriptional regulation of *SNAI2* does not require binding to CRL4s. Thus, AMBRA1 targeting into the nucleus is likely dependent on other factors. It is important to note that we cannot rule out the possibility that AMBRA1 indirectly regulates transcription by binding and sequestering transcriptional regulators in the cytosol.

What are the genes regulated by AMBRA1? Gene Ontology enrichment analysis of differentially expressed genes revealed that many affected genes are involved in extracellular matrix composition and remodeling, as well as cell-substrate adhesion. Indeed, previous evidence indicates that AMBRA1 can be recruited to FAs, where it controls the levels of active Src and FAK (Schoenherr et al., 2017). We found that mRNA and protein levels of paxillin, a FA adapter protein, were significantly increased in AMBRA1-KO cells. In contrast, the mRNA levels of *SNAI2*, encoding a zinc-finger transcription factor that is involved in EMTs, were significantly decreased. All these findings suggest that regulation of gene transcription by AMBRA1 affects cell migration. Consistent with this hypothesis, we observed that AMBRA1 depletion affected the size and position of FAs, as well as the speed and directionality of cell migration. Importantly, we observed that MDA-MB-231 TKO cells (lacking Rab40a, Rab40b and Rab40c) exhibited similar effects on cell migration, further supporting the hypothesis that Rab40-dependent ubiquitylation enhances the transcriptional effects of AMBRA1.

AMBRA1 is a multifunctional protein that is involved in many cellular processes, including autophagy, proliferation, apoptosis, transcription and cancer drug resistance (Chen et al., 2023; Cianfanelli et al., 2015a; Di Leo et al., 2024; Di Rienzo et al., 2024; Fimia et al., 2013; Frias et al., 2023; Sun, 2016). Here, we examined the potential roles of AMBRA1 in regulating transcription and cell migration. Furthermore, we show that Rab40–CRL5-dependent ubiquitylation of AMBRA1 appears to be required for its transcriptional function. However, many questions remain. We do not know what AMBRA1 amino acid residues are ubiquitylated by Rab40–CRL5 and what type of ubiquitylation (Lys63 or Lys48) is mediated by Rab40–CRL5. It is also unclear whether Rab40–CRL5-dependent ubiquitylation also affects canonical AMBRA1 function, such as regulating autophagy. Finally, we are only beginning to understand how AMBRA1 regulates transcription, and what the functions are of different AMBRA1 splice isoforms. Further studies will be needed to address all these questions.

## MATERIALS AND METHODS

### Cell culture and transfection

All cell lines were cultured as described previously (Duncan et al., 2022; Han et al., 2016, 2022). Briefly, human embryonic kidney (HEK) 293T cells were grown in complete DMEM [Dulbecco's modified Eagle medium (DMEM; Mediatech, 10-017-CV) supplemented with 10% fetal bovine serum (Phoenix Scientific, ps-100) and 100 μg/ml of penicillin and streptomycin] at 37°C in a 5% CO_2_ atmosphere. MDA-MB-231 cells were grown in complete DMEM supplemented with 1 µg/ml human recombinant insulin (Gibco, 12585-014), 1% non-essential amino acids (Gibco, 11140-050) and 1% sodium pyruvate. Cell lines were routinely tested for mycoplasma. All cell lines used in this study were authenticated and are in accordance with American Type Culture Collection standards. 293T cells were grown to 60–70% confluence and transfected using the standard calcium phosphate precipitation method (Han et al., 2016, 2012). Typically, 10 μg of plasmid was used for a single-gene transfection of a 100-mm dish of cells, with up to 30 μg of plasmids used for co-transfection of three plasmids. MDA-MB-231 cells were grown to 80–90% confluence and transfected using jetPRIME (Polyplus). Lipofectamine RNAiMAX (Invitrogen) was used for transfection of siRNAs both in 293T and MDA-MB-231 cells.

### Antibodies and reagents

The following antibodies were used in this study: anti-FLAG [clone M2; #F1804, 1:1000 for western blotting (WB)] from Sigma-Aldrich; anti-paxillin [#610620, 1:500 for immunofluorescence (IF)] from Transduction Laboratories; anti-GFP (GF28R; #Y1031, 1:2000 for WB) from UBPBio; rabbit anti-AMBRA1 (#13762-1-AP, 1:1000 for WB), rabbit anti-cullin-4A (#14851-1-AP, 1:2000 for WB) and rabbit anti-cullin-4B (#12916-1-AP, 1:2000 for WB) from Proteintech; mouse anti-c-Myc (9E10, #sc-40, 1:1000 for WB), mouse anti-HA (F-7, #sc-7392, 1:500 for WB), mouse anti-α-tubulin (sc-23948, 1:3000 for WB), anti-Rab40c (H-8, #sc-514826, 1:500 for WB), anti-cullin-5 (H-300, #sc-13014, 1:500 for WB) and mouse anti-AMBRA1 (G-6, #sc-398204, 1:500 for WB) from Santa Cruz Biotechnology; rabbit anti-cyclin D1 (#E395S, 1:1000 for WB), rabbit anti-SNAI2 (C19G7; #9585, 1:1000 for WB), rabbit anti-HP1 (#2616, 1:1000 for WB) from Cell Signaling Technology. Specificity of anti-AMBRA1 antibodies was confirmed by immunoblotting lysates from control and AMBRA1 siRNA knockdown cells (Fig. S1A).

TRIzol, puromycin, phalloidin–Alexa Fluor 594 (A12381, 1:1000 for IF) and dox were purchased from Thermo Fisher Scientific. MG132 and BFM (S1413) were purchased from Selleckchem. Complete protease inhibitor cocktail and phosphatase inhibitor cocktails were purchased from Roche.

### Mammalian expression constructs

Plasmids expressing human Rab40a, Rab40al, Rab40b, Rab40c, HA–Rab40c, the FLAG–Rab40c-4A mutant and Myc–Ub have been described previously (Han et al., 2022). pcDNA4-AMBRA1-3xFLAG (#174157) and pCW-Cas9 (#50661) were obtained from Addgene. The plasmid expressing AMBRA1 isoform1 was purchased from GeneCopoeia. AMBRA1 isoform 5 was cloned by PCR. GFP–AMBRA1, and the GFP–AMBRA1(D51–200), HA–AMBRA1-ΔN (1–20 deletion) and FLAG–AMBRA1 deletion mutants were constructed by PCR, followed by subcloning into the pRK7 (Addgene plasmid #10883) or pCW (from pCW-Cas9) vector containing an N-terminal GFP or FLAG tag. All plasmids were validated by DNA sequencing.

### Immunoprecipitation and western blot analysis

For non-denaturing immunoprecipitation, cells in a 100-mm dish were harvested and lysed on ice in buffer containing 20 mM Tris-HCl, pH 7.4, 150 mM NaCl, 2 mM EDTA, 1% Triton X-100, 10% glycerol with protease inhibitor, and phosphatase inhibitor cocktails. After clearing lysates by centrifugation, supernatants were incubated with 2 μg of an appropriate antibody or control IgG (mouse IgG, Sigma, I5381; rabbit IgG, Sigma, I5006) for 4 h at 4°C, then supplemented with 50 μl protein G beads (Cytiva, #17061801). After overnight incubation, the protein G beads were pelleted by centrifugation and washed three times with 1 ml of lysis buffer plus 0.5 M NaCl. Bound proteins were eluted in 50 μl 1× SDS sample buffer. For denaturing immunoprecipitation, cells in a 100-mm dish were lysed in 1 ml cell lysis buffer plus 1% SDS. Cell lysates were collected and then heated at 95°C for 10 min. After centrifugation, supernatants were diluted with the cell lysis buffer to reduce the SDS concentration to 0.1%. The immunoprecipitation assay was performed as described above, except that 5 μg anti-FLAG M2 antibody was used in each reaction. Eluates were then resolved by SDS-PAGE and transferred to nitrocellulose membranes for immunoblotting assays. Immunoblotting images were captured using a ChemiDoc MP Imaging system (Bio-Rad).

### MDA-MB-231 CRISPR/Cas9 KO cell lines and genotyping

MDA-MB-231 cells stably expressing tetracycline-inducible Cas9 (Horizon Discovery, Edit-R lentiviral Cas9) were grown in a 12-well plate to ∼75% confluency and then treated with 1 µg/ml dox for 24 h to induce Cas9 expression. After 24 h, cells were co-transfected with a crRNA:tracrRNA mix using DharmaFECT Duo transfection reagent as described by the Horizon Discovery DharmaFECT Duo protocol. The crRNAs for AMBRA1 targeting were 5′-TACCATTACTGATTTCAGGG-3′ (exon 12, isoform 2) and 5′-ACTGACATGTCTCCGCTGGT-3′ (exon 16, isoform 2). Cells were split 24 h after transfection and seeded as single colonies. They were then screened by western blotting, followed by PCR cloning and genotyping. Rab40a-KO, Rab40b-KO, Rab40c-KO and Rab40-TKO lines have been described previously (Linklater et al., 2021). For each KO line, two different clonal lines were used for experiments, and their mRNA relative expression levels were also quantified by qPCR (Fig. S1C).

### qPCR

Total RNA was extracted using TRIzol according to the manufacturer's protocol. Reverse transcription to cDNA was performed with SuperScript IV (Invitrogen) using oligo(dT) primers. qPCR was performed using iTaq SYBR Green qPCR Master Mix (BIO-RAD, #1725121) on an Applied Biosystems ViiA7 real-time PCR system. The qPCR amplification conditions were 50°C (2 min), 95°C (10 min), 40 cycles at 95°C (15 s), and 60°C (1 min). Targets were normalized to *GAPDH* levels. The following primers used for qPCR were from PrimerBank (https://pga.mgh.harvard.edu/primerbank/): *RAB40C*, forward, 5′-GGCCCAACCGAGTGTTCAG-3′, and reverse, 5′-GGACTTGGACCTCTTGAGGC-3′; *AMBRA1*, forward, 5′-CTCTTCCTCAGACAACCAGGGT-3′, and reverse, 5′-TCCAAGCGAAGGTGCAGACATC-3′; paxillin, forward, 5′-ACAGTCGCCAAAGGAGTCTG-3′, and reverse, 5′-GGGGCCGTTGCAGTAGTAG-3′; *MAP4K4*, forward, 5′-GGAACACACTCAAAGAAGACTGG-3′, and reverse, 5′-GTGCCTATGAACGTATTTCTCCG-3′; *GRAMD1B*, forward, 5′-GCTATGGGAACGAATTGGGC-3′, and reverse, 5′-CTGCTCTTGGATGAGCTGTCA-3′; *SNAI2*, forward, 5′-TGTGACAAGGAATATGTGAGCC-3′, and reverse, 5′-TGAGCCCTCAGATTTGACCTG-3′; *PXDN*, forward, 5′-AATCAGAGAGATCCAACCTGGG-3′, and reverse, 5′-AATGCTCCACTAGGTATCCTCTT-3′; and *GAPDH*, forward, 5′-CTGGGCTACACTGAGCACC-3′, and reverse, 5′-AAGTGGTCGTTGAGGGCAATG-3′.

### Cellular fractionation

Cytoplasmic and nuclear or cytoskeletal fractions were isolated using the Cell Fractionation Kit from Cell Signaling Technology (#9038) according to the manufacturer's instructions. Briefly, MDA-MB-231 cells at 100% confluency in a 10 cm dish were trypsinized and washed in PBS. The cell pellets were resuspended in 500 µl Cytoplasmic Isolation Buffer, vortexed for 5 s, incubated on ice for 5 min, and centrifuged for 5 min at 500 ***g***. The supernatant was saved as the cytoplasmic fraction. The pellet was resuspended in 500 µl Membrane Isolation Buffer, incubated on ice for 5 min, and centrifuged for 5 min at 8000 ***g*** to remove the supernatant (the membrane and organelle fraction). The pellet was resuspended directly in 50 µl 1× SDS as the cytoskeletal and nuclear fraction.

### *In vivo* ubiquitylation assay

*In vivo* ubiquitylation assay was performed as described previously (Duncan et al., 2022; Han et al., 2016; Linklater et al., 2021). Briefly, 293T cells (∼70% confluency) were transfected with various combinations of plasmids including Myc–Ub. After 24 h, cells were treated with 10 μM MG132 for 6 h or 100 nM BFM overnight. Then, cells were lysed in 1% SDS for denaturing immunoprecipitation as described above. Bound proteins were eluted in 50 µl 1× SDS sample buffer. Eluates (20 µl) were resolved via SDS-PAGE and transferred to nitrocellulose membranes for immunoblotting.

### siRNA knockdown

AMBRA1 siRNAs (SASI_Hs01_00116731 and SASI_Hs01_00116732) and mission siRNA universal negative control (SIC001; Sigma) were purchased from Sigma-Aldrich. siRNAs were transfected using Lipofectamine RNAiMAX (Invitrogen) according to the manufacturer's protocol.

### RNA-seq

RNA-seq was performed by the National Jewish Health Genomics Facility. Briefly, total RNA was isolated using TRIzol (Invitrogen) according to the manufacturer's protocol. RNA-seq libraries were prepared according to the KAPA mRNA HyperPrep Library Build user guide (Roche; https://rochesequencingstore.com/wp-content/uploads/2017/10/KAPA-mRNA-HyperPrep-Kit_KR1352-%E2%80%93-v5.17.pdf). mRNA from 50 ng of total RNA was isolated using polyA, oligo-dT magnetic beads (Roche, KK8580). The isolated mRNA was then subject to enzymatic fragmentation, resulting in 200–300 bp fragments. The resulting RNA fragments underwent first- and second-strand cDNA synthesis. Unique KAPA Dual Indexes (Roche, KK8727) were then ligated to the cDNA. The ligated product was then PCR amplified for 13 cycles. The resulting libraries were quantified using the Qubit HSDNA assay (Invitrogen, Q32854) and the TapeStation HSDNA 1000 assay (Agilent, 5067-5584). Equal molar concentrations were pooled, diluted and sequenced using a NovaSeq 6000 sequencing system (Illumina).

### Flow cytometry

Flow cytometry was used for cell cycle analysis, which was conducted by CU Cancer Center Flow Cytometry Shared Resource at the University of Colorado. MDA-MD-231 Cas9 control and two AMBRA1-KO cell lines were stained by propidium iodide as described previously (Krishan, 1975). Cells were analyzed using a Beckman Coulter Gallios flow cytometer. Doublets were excluded from the analysis using the peak versus integral gating method. ModFit LT software (Verity Software House, Topsham, ME, USA) was used for cell cycle analysis.

### Cell migration assays

For single-cell migration assays, time-lapse imaging was performed using an OLYMPUS IX83 inverted confocal microscope, with a brightfield 4× magnification objective equipped with a humidified chamber and temperature-controlled stage top. 35 mm glass-bottomed dishes were coated with fibronectin (F4759-1mg, Sigma-Aldrich) and allowed to set for 1 h under ultraviolet light at room temperature. Once dried, the cells were plated and allowed to attach for 24 h. All time-lapse images were taken at 20 min intervals and 36 frames were taken, resulting in a total time-lapse duration of ∼12 h. For cell migration analysis, cells were manually tracked using the Manual Tracking software Excellence Pro (Olympus Europe). Generated data were acquired from this software, such as speed, which was used to calculate velocity. Careful effort was made to select the geometric center of the cell to focus on the nucleus when manually tracking. Three independent biological replicates were performed for each cell line. Statistical analysis (non-parametric two-tailed unpaired Student's *t*-test) and Mann–Whitney test were performed on the averages of the three biological replicates and processed using GraphPad Prism.

### Immunofluorescence microscopy and image analysis

MDA-MB-231 cells were seeded onto collagen-coated glass coverslips and grown in full growth medium unless otherwise noted for at least 24 h. Cells were washed with PBS and fixed in 4% paraformaldehyde for 15 min. Samples were then washed three times in PBS, then incubated in blocking serum (1× PBS, 5% normal donkey serum and 0.3% Triton X-100) for 1 h at room temperature. Primary antibodies were then diluted at 1:100 in dilution buffer (1× PBS, 1% BSA and 0.3% Triton X-100) and incubated for 1 h at room temperature. Samples were then washed three times with PBS and incubated with fluorophore-conjugated secondary antibodies (1:100 in dilution buffer) for 30 min at room temperature. Cells were then washed three times in PBS and mounted onto glass slides. Cells were then imaged on either an inverted Zeiss Axiovert 200M deconvolution microscope with a 63× oil-immersion lens and Sensicam QE charge-coupled device camera, or a Nikon A1R confocal microscope. *Z*-stack images were taken at a step size of 100–500 nm.

Analysis of FA number and quantification of FA size were performed in ImageJ and were adapted from Horzum et al. (2014). For each experimental replicate, cells were analyzed from at least five randomly chosen fields. In total, 15–20 cells were analyzed for each experimental replicate. Only cells that did not make any contact with the surrounding cells were analyzed. The same exposure was used for all images for each experimental replicate. Maximum-intensity projections for relevant *z*-planes were created, and images were loaded into ImageJ. The background was minimized using the ‘Subtract Background’ and ‘EXP’ tools, images were filtered using the Log3D and Mexican Hat Filter plugin, and thresholds were then applied manually (method=default). Individual cells were defined by hand, and FAs were determined with the ‘Analyze Particle’ command. The resulting particle outlines were then compared with the original image to ensure fidelity of the analysis. To analyze the number of FAs in the cell periphery, the 4-µm-wide area of interest around the cell was selected at the plasma membrane. The FAs in that area were then counted and the ratio of FAs in the periphery to FAs in the whole cell was calculated for each individual cell.

### Statistical analysis

Statistical analysis for all experiments was determined using GraphPad Prism software. A two-tailed unpaired Student's *t*-test was used to determine statistical significance unless otherwise noted. Data were collected from at least three independent experiments unless otherwise noted. In all cases, *P*≤0.05 was regarded as significant. Error bars represent standard errors unless otherwise noted. For all immunofluorescence experiments, at least five randomly chosen image fields per condition were used for each experimental replicate. In total, 15–20 cells were analyzed for each experimental replicate, and each individual cell was treated as a technical replicate. Statistical analysis was performed on means calculated from individual cells for each experimental replicate.

## Supplementary Material

10.1242/joces.263707_sup1 Supplementary information
