# Supplementary material for: Rab40 GTPases regulate AMBRA1-mediated transcription and cell migration
Source: J Cell Sci. 2025 Apr 11;138(7):jcs263707. doi: 10.1242/jcs.263707 (PMC12045048; doi:10.1242/jcs.263707)
Supplement: Supplementary information [file joces-138-263707-s1.pdf]

A

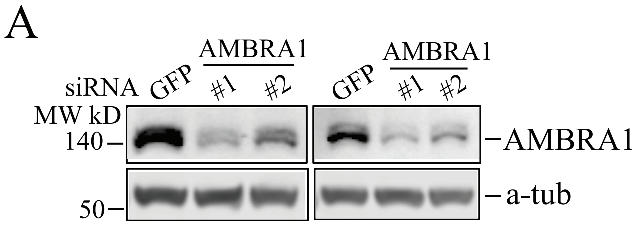

C

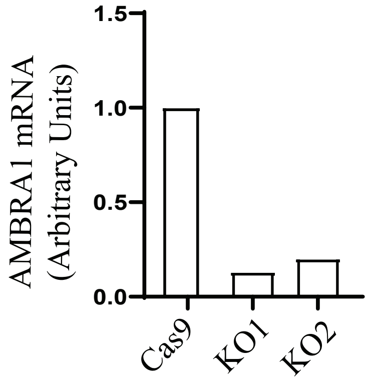

B

Clone A9 heterogeneous, allele#1: GGG ACT TCA CTA AGT TTG ACC TCC C 19bp deletion causes a premature stop codon

MKVVPEKNAVRILWGRERGARAMGAQRLLQELVEDKTRWMKWEGKRVELPDSPRSTFLLAFSPDRTLLA
STHVNHNIYITEVKTGKCVHSLIGHRRTPWCVTFHPTISGLIASGCLDGEVRIWDLHGGSESWFTDSNNAIAS
LAFHPTAQLLLIATANEIHFWDWSRREPFAVVKTASEMERVRLVRFDPLGHYLLTAIVNPSNQQGDDEPEIP
IDGTELSHYRQRALLQSQPVRRTPLLHNFLHMLSSRSSGIQTEPFHPPEQASSTQQDQGLLNRPSAFSTVQSS
TAGNTLRNLSLGPTRRSLGGPLSSHPSRYHREIAPGLTGSEWTRTVLSLNSRSEAESMPPPRTSASSVSLLSV
LRQQEGGSQASVYTSATEGRGFPASGLATESDGGNGSSQNNSGSIRHELQCDLRRFFLEYDRLQELDQSLS
GEAPQTQQAQEMLNNNIESERPGPSHQPTPHSSENNSNLSRGHLNRCRACHNLLTFNNDTLRWERTTPNYS
SGEASSSWQVPSSFESVPSSGSQLPPLERTEGQTPSSSRLELSSSASPQEERTVGVAFNQETGHWERIYTQSSR
SGTVSQEALHQDMPEESSEEDSLRRRSLALSPRLEYSGAILAHCKLRLPGSCHSPASASQVAGTTGAHHHAR
LIFAFLVEMEFHHVSQAGLELLTSGDLPTSASQSAGITGVSHRAWPRLLESSLISLSRYDGAGSREHPIYPDP
ARLSPAAYYAQRMIQYLSRRDSIRQRSMRYQQNRLRSSTSSSSSDNQGPSVEGTDLEFEDFEDNGDRSRHR
APRNARMSAPSLGRFVPRRFLLPEYLPYAGIFHERGQPGLATHSSVNRVLAGAVIGDGQSAVASNIANTTY
RLQW(867)LKSVMLP-STOP

allele #2: CCCTGAAATCAGTAAT 16bp deletion causes a premature stop codon

MKVVPEKNAVRILWGRERGARAMGAQRLLQELVEDKTRWMKWEGKRVELPDSPRSTFLLAFSP
DRTLLASTHVNHNIYITEVKTGKCVHSLIGHRRTPWCVTFHPTISGLIASGCLDGEVRIWDLHGGSE
SWFTDSNNAIASLAFHPTAQLLLIATANEIHFWDWSRREPFAVVKTASEMERVRLVRFDPLGHYLL
TAIVNPSNQQGDDEPEIPIDGTELSHYRQRALLQSQPVRRTPLLHNFLHMLSSRSSGIQTEPFHPPEQ
ASSTQQDQGLLNRPSAFSTVQSSTAGNTLRNLSLGPTRRSLGGPLSSHPSRYHREIAPGLTGSEWTR
TVLSLNSRSEAESMPPPRTSASSVSLLSVLRQQEGGSQASVYTSATEGRGFPASGLATESDGGNGSS
QNNSGSIRHELQCDLRRFFLEYDRLQELDQSLSGEAPQTQQAQEMLNNNIESERPGPSHQPTPHSSE
NNSNLSRGHLNRCRACHNLLTFNNDTLRWERTTPNYSSGEASSSWQVPSSFESVPSSGSQLPPLER
TEGQTPSSSRLELSSSASPQEERTVGVAFNQETGHWERIYTQSSRSGTVSQEALHQDMPEESSEEDS
LRRRSLALSPRLEYSGAILAHCKLRLPGSCHSPASASQVAGTTGAHHHARLIFAFLVEMEFHHVSQ
AGLELLTSGDLPTSASQSAGITGVSHRAWPRLLESSLISLSRYDGAGSREHPIYPDPARLSPAAYYA
QRMIQYLSRRDSIRQRSMRYQQNRLRSSTSSSSSDNQGPSVEGTDLEFEDFEDNGDRSRHRAPRNA
RMSAPSLGRFVPRRFLLPEYLPYAGIFHERGQPGLATHSSVNRVLAGAVIGDGQSAVASNIANTTY
RLQWWDFTKFDL(877)LP-STOP

Clone B6 homogeneous GCCTGCCGAC<u>C</u>AGCGGAGACAT 1bp C deletion causes a premature stop codon

MKVVPEKNAVRILWGRERGARAMGAQRLLQELVEDKTRWMKWEGKRVELPDSPRSTFLLAFSPDRTLLA
STHVNHNIYITEVKTGKCVHSLIGHRRTPWCVTFHPTISGLIASGCLDGEVRIWDLHGGSESWFTDSNNAIAS
LAFHPTAQLLLIATANEIHFWDWSRREPFAVVKTASEMERVRLVRFDPLGHYLLTAIVNPSNQQGDDEPEIP
IDGTELSHYRQRALLQSQPVRRTPLLHNFLHMLSSRSSGIQTEPFHPPEQASSTQQDQGLLNRPSAFSTVQSS
TAGNTLRNLSLGPTRRSLGGPLSSHPSRYHREIAPGLTGSEWTRTVLSLNSRSEAESMPPPRTSASSVSLLSV
LRQQEGGSQASVYTSATEGRGFPASGLATESDGGNGSSQNNSGSIRHELQCDLRRFFLEYDRLQELDQSLS
GEAPQTQQAQEMLNNNIESERPGPSHQPTPHSSENNSNLSRGHLNRCRACHNLLTFNNDTLRWERTTPNYS
SGEASSSWQVPSSFESVPSSGSQLPPLERTEGQTPSSSRLELSSSASPQEERTVGVAFNQETGHWERIYTQSSR
SGTVSQEALHQDMPEESSEEDSLRRRSLALSPRLEYSGAILAHCKLRLPGSCHSPASASQVAGTTGAHHHAR
LIFAFLVEMEFHHVSQAGLELLTSGDLPTSASQSAGITGVSHRAWPRLLESSLISLSRYDGAGSREHPIYPDP
ARLSPAAYYAQRMIQYLSRRDSIRQRSMRYQQNRLRSSTSSSSSDNQGPSVEGTDLEFEDFEDNGDRSRHR
APRNARMSAPSLGRFVPRRFLLPEYLPYAGIFHERGQPGLATHSSVNRVLAGAVIGDGQSAVASNIANTTY
RLQWWDFTKFDLPEISNASVNVLVQNCKIYNDASCDISADGQLLAAFIPSSQRGFPDEGILAVYSLAPHNLG
EMLYTKRFGPNAISVSLSPMGRYVMVGLASRRILLHPSTEHMVAQVFRLQQAHGGETSMRRVFNVLYPMP
AD(1007)SGDMSVSTLPVGCLSQGLAWPMVLTKETW-STOP

Journal of Cell Science • Supplementary information

Fig. S1. AMBRA1 antibody confirmation and AMBRA1 KO cell genotyping.

(A) To test the specificity of the anti-AMBRA1 antibody, MDA-MB-231 cells were transfected with non-targeting control siRNA or siRNA targeting AMBRA1. Cell lysates were then blotted with anti-AMBRA1 or anti-α-tubulin antibodies.

(B) Genotyping two of AMBRA1 MDA-MB-231 cell lines. Deletions are underlined. Predicted amino acids are shown under the deleted nucleotide sequences. Extra introduced amino acids by the frame shift are highlighted in red. (C) qPCR analysis of AMBRA1 mRNA levels in control and AMBRA1-KO MDA-MB-231 cells.

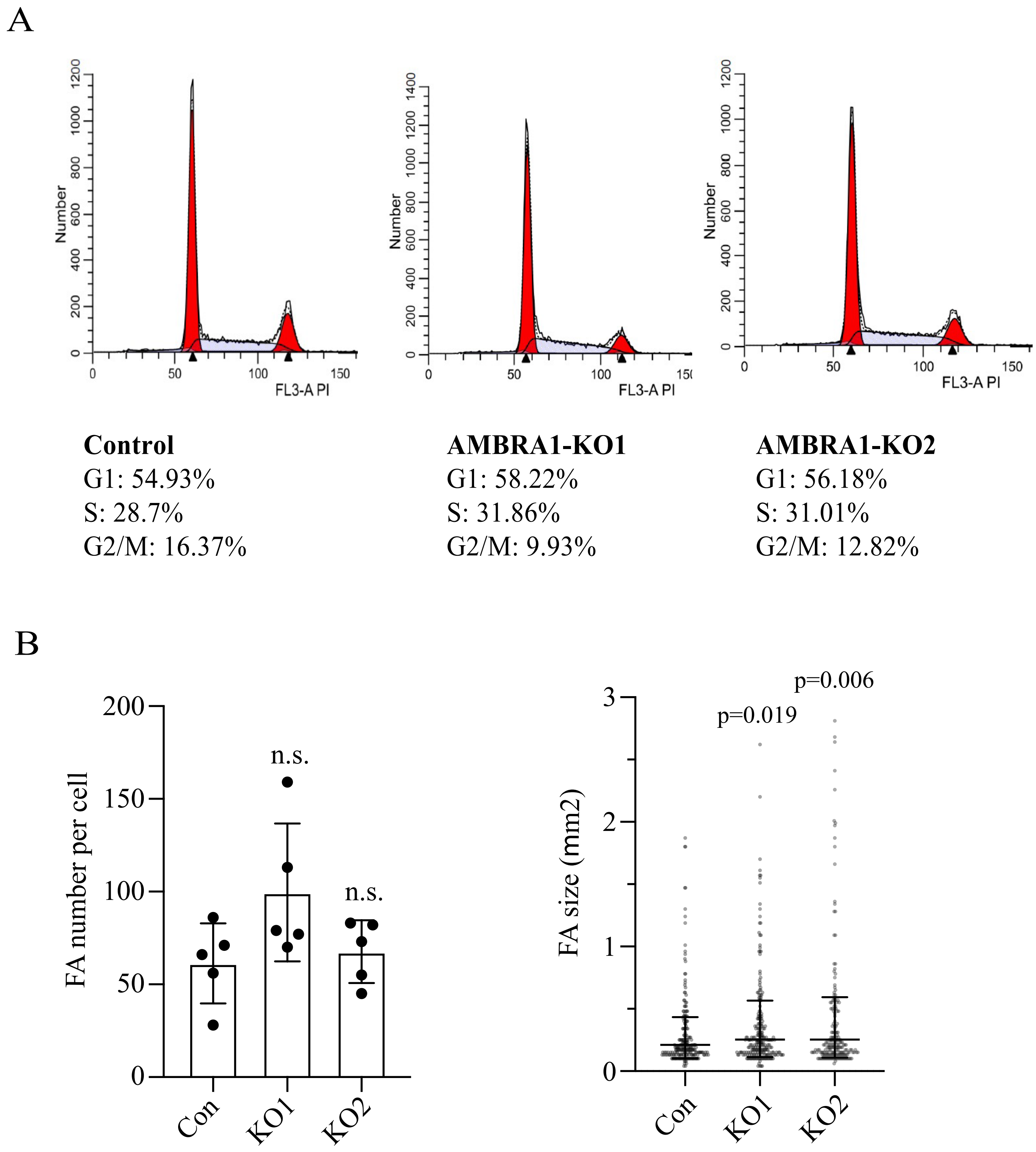

Fig. S2. Cell cycle distribution and FA number and size of control and AMBRA1 KO cells.
(A) Control and AMBRA1-KO MDA-MB-231 cells were analyzed for DNA content by flow cytometry. Cells were fixed, permeabilized, and stained with propidium iodide (PI) and the cell cycle distribution of each cell type was performed by flow cytometry (see Materials and methods). Quantitative cell cycle phases proportions were identified by calculating the cell number % of each cell cycle phase relative to total phases after appropriate gating of cell populations by PI fluorescence and showed under the histogram of DNA content distribution.
(B) Quantification of number (left) and size (right) of FAs in control and AMBRA1-KO cells. Data shown are means and SDs derived from three independent experiments.

Journal of Cell Science • Supplementary information

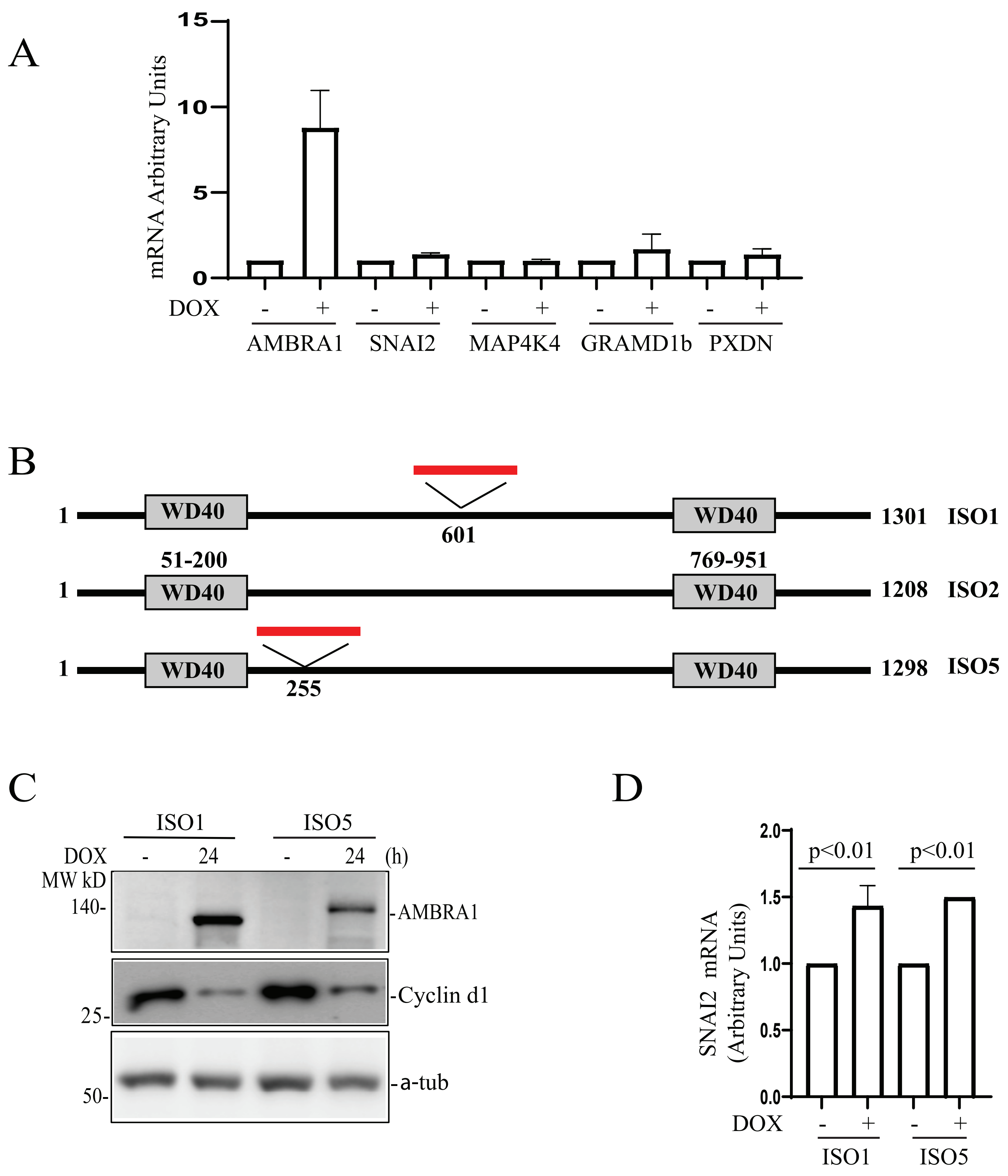

Fig. S3. Effects of Rab40 KO and AMBRA1 isoforms on AMBRA1 mediated transcription.

(A) qRT-PCR analysis of the levels of selected mRNA levels in Rab40-KO MDA-MB-231 cells stably expressing dox-inducible AMBRA1.

(B) A schematic representation of AMBRA1 isoforms 1, 2, and 5. The short lines in red represent insertions.

(C) AMBRA1-KO MDA-MB-231 cells stably expressing dox-inducible AMBRA1 isoforms 1 or 5 were incubated with 100 ng/ml doxycycline for 48h. Cell lysates were immunoblotted with anti-AMBRA1, anti-cyclin d1, or anti-α-tubulin antibodies.

(D) qRT-PCR analysis of the levels of SNAI2 mRNA in AMBRA1-KO MDA-MB-231 cells stably expressing either dox-inducible AMBRA1 isoform 1 or 5. The means and SD were calculated from three independent experiments.

Journal of Cell Science • Supplementary information

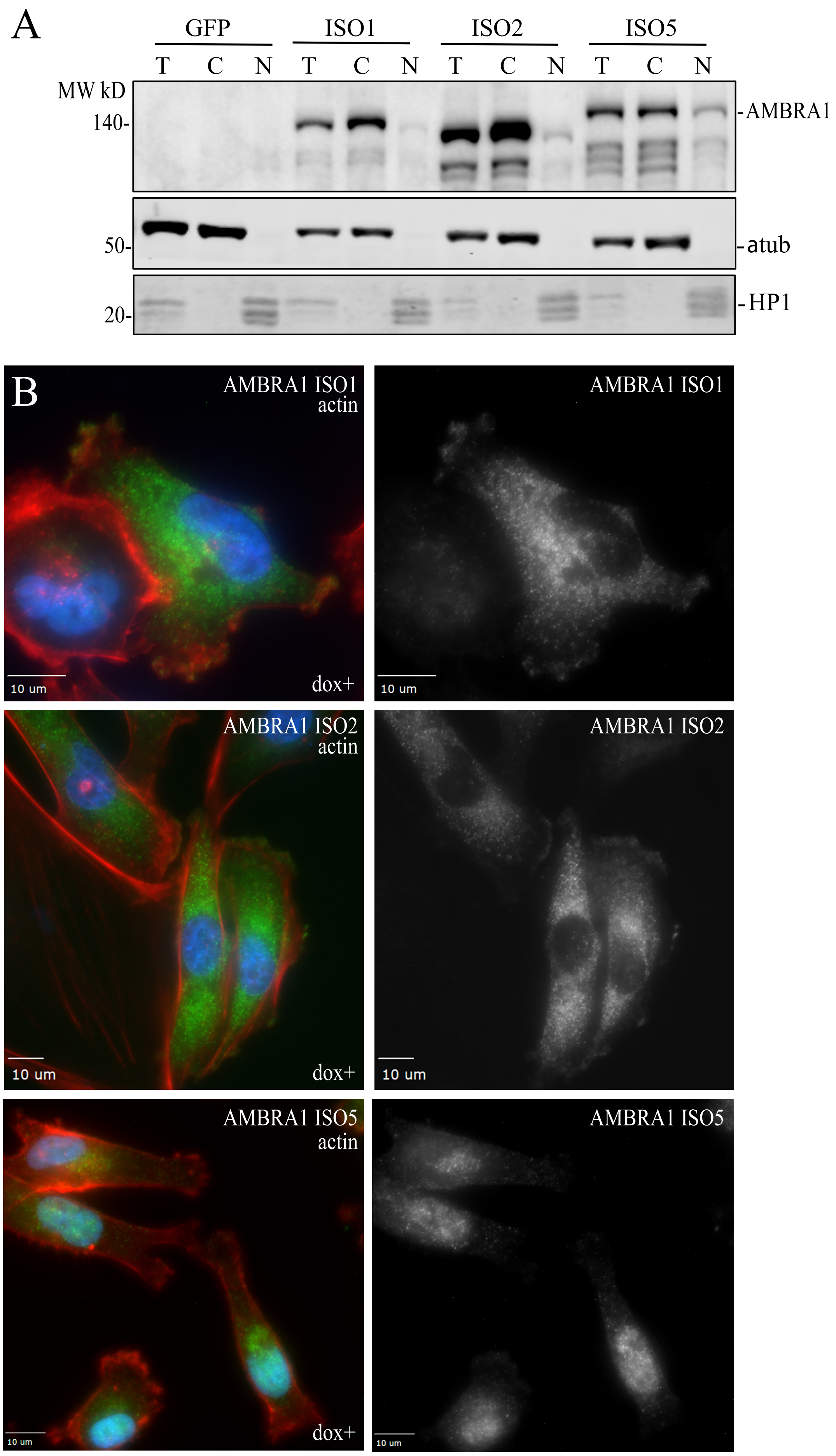

Fig. S4. Subcellular localization of AMBRA1 isoforms.

(A) AMBRA1-KO MDA-MB-231 cells stably expressing dox-inducible GFP, AMBRA1 isoforms 1, 2, or 5 were incubated with 100 ng/ml doxycycline for 48h. Total (T), cytoplasmic (C), and nuclear (N) fractions were collected (see Materials and methods) and subjected to Western blot by indicated antibodies.

(B) AMBRA1-KO MDA-MB-231 cells stably expressing dox-inducible AMBRA1 isoforms 1, 2, or 5 were plated on collagen-coated coverslips for 24 hours and then were incubated with 100 ng/ml doxycycline for 24h. Cells were then fixed and stained with phalloidin-Alexa Fluor 594 (red) and anti-AMBRA1 antibodies (green).
